# Tryptase-Positive Mast Cells Promote Adipose Fibrosis in Secondary Lymphedema through PDGF

**DOI:** 10.3390/cimb45100507

**Published:** 2023-09-30

**Authors:** Takashi Nuri, Denan Jin, Shinji Takai, Koichi Ueda

**Affiliations:** 1 Department of Plastic and Reconstructive Surgery, Osaka Medical and Pharmaceutical University, Takatsuki 569-8686, Japan; koichi.ueda@ompu.ac.jp; 2 Department of Innovative Medicine, Osaka Medical and Pharmaceutical University, Takatsuki 569-8686, Japan; denan.jin@ompu.ac.jp (D.J.); shinji.takai@ompu.ac.jp (S.T.)

**Keywords:** tryptase, mast cells, secondary lymphedema, adipose fibrosis, platelet-derived growth factor A (PDGF-A)

## Abstract

Lymphedema is a chronic and progressive condition that causes physical disfigurement and psychological trauma due to the accumulation of lymphatic fluid in the interstitial space. Once it develops, lymphedema is difficult to treat because it leads to the fibrosis of adipose tissue. However, the mechanism behind this remains unclear. The purpose of this study was to investigate the involvement of mast cells (MCs) in the adipose tissues of patients with lymphedema. We found that fibrosis spread through blood vessels in the adipose tissues of lymphedema patients, and the expression of the collagen I and III genes was significantly increased compared to that of those in normal adipose tissue. Immunostaining of vimentin and α-smooth muscle actin showed that fibroblasts were the main cellular components in severely fibrotic regions. Toluidine blue staining confirmed a significant increase in the number of MCs in the adipose tissues of lymphedema patients, and immunostaining of serial sections of adipose tissue showed a significant increase in the number of tryptase-positive cells in lymphedema tissues compared with those in normal adipose tissues. Linear regression analyses revealed significant positive correlations between tryptase and the expressions of the TNF-α, platelet-derived growth factor (PDGF)-A, and PDGFR-α genes. PDGF-A–positive staining was observed in both fibroblasts and granules of tryptase-positive MCs. These results suggest that MC-derived tryptase plays a role in the fibrosis of adipose tissue due to lymphedema directly or in cooperation with other mediators.

## 1. Introduction

Lymphedema is a debilitating condition that causes physical disfigurement and psychological trauma. Secondary lymphedema can occur after cancer treatment involving lymph node dissection or radiation therapy. Most lymphedema patients from developed countries exhibit this condition due to treatments for malignancy. Previous studies reported that lower-limb lymphedema occurs in 28–47% of gynecologic patients who undergo lymph node dissection [1,2], and upper-limb lymphedema occurs in 33% of breast cancer patients as a result of the same procedure [3].

The International Society of Lymphedema staging is a well-known three-stage scale for classifying lymphedematous limbs [4]. Stage 0 refers to a latent or sub-clinical condition in which swelling is not yet evident despite impaired lymph transport, but subtle changes in tissue fluids and subjective symptoms are observed. Stage I represents the early accumulation of fluid that is relatively high in protein content, which subsides with limb elevation. An increase in various types of proliferating cells may also be witnessed after this stage. In Stage II, limb elevation alone rarely reduces tissue swelling. In Late Stages II and III, the extremities may not pit, due to skin thickening and further deposition of adipose tissue.

Previous studies have demonstrated that fibrosis is both a phenotypic and pathogenic factor in lymphedema, and analyses of clinical samples have revealed that lymphedema causes smooth muscle cell growth, lymphatic channel luminal blockage, and progressive collecting lymphatic vessel fibrosis [5,6]. Adipose tissue fibrosis includes the accumulation and increased production of extracellular matrix proteins. In healthy adipose tissue, the extracellular matrix can be remodeled to accommodate normal fluctuations in adipocyte size. However, when adipose tissue becomes fibrotic, the stiff extracellular matrix cannot be dynamically remodeled [7]. In lymphedema, fibro-adipose tissue deposition is also identified [8,9]. Although fibroblast proliferation is crucial to tissue regeneration and repair, excessive fibrous connective tissue development compromises tissue architecture and function. The collecting lymphatic vessels are primarily located in the subcutaneous adipose layer, and deposition of the fibro-adipose tissue further aggravates lymphatic flow and interferes with lymphatic vascular flow. Once the fibro-adipose tissue develops, the treatments aimed at draining accumulated lymph fluid, i.e., lymphaticovenular anastomosis and manual lymph drainage, are no longer effective. Suction-assisted lipectomy is one option for adipose tissue accumulation. However, in cases of severe fibrosis, direct excision becomes necessary [10,11].

In fibrosis-affecting tissues such as the liver [12], lungs [13], and pancreas [14], mast cells (MCs) are observed in the fibrous region. Previous studies showed that the number of MCs increased in the dermis of the skin in fibrosis in Stages II and III lymphedema [15]. These MCs promote the proliferation of fibroblasts and the formation of collagen by secreting a variety of mediators, including tumor necrosis factor α (TNF-α). Fibrosis in the adipose tissues has also been reported in obese individuals, and MCs have been observed in the adipose tissue of these individuals, especially in fibrosis bundles [16,17]. These data suggest that MCs contribute to the fibrosis of adipose tissues in lymphedema patients.

The aim of this study was to compare the expression characteristics of MCs and the regulating fibrogenic mediators in adipose tissues obtained from patients with secondary lymphedema.

## 2. Materials and Methods

### 2.1. Sample Collection

Adipose tissues were collected from the thighs of 17 patients with secondary lower extremity lymphedema (16 females and 1 male; mean age, 66 years; age range, 46–79 years; mean BMI (body mass index), 23.6; BMI range, 14.9–34). In all female patients, secondary lymphedema developed after treatment for gynecological cancer. In the male patient, secondary lymphedema developed after treatment for bladder cancer. The average period since primary operation for cancer treatment was 13.8 years (range, 3–26 years). The diagnosis of lymphedema was confirmed using radio isotope lymphoscintigraphy. The images were observed 15–20 min (early phase) and 120 min (late phase) after tracer injection. The diagnosis of lymphedema was obtained from imaging findings of dermal back flow. The numbers of patients in each ISL (International Society of Lymphedema) score were identified as follows: Stage II—12, Late Stage II—4, and Stage III—1. Normal control samples were obtained from the thighs (*n* = 4) and lower leg (*n* = 1) of 5 patients (2 females and 3 males; mean age, 64 years; age range, 48–76 years; mean BMI, 18.1) who underwent reconstruction of the head and neck using the anterolateral thigh free flap and fibula flap to treat oral and maxillofacial cancer. Adipose tissues were obtained when the size of the skin flap was adjusted to the defect. Adipose tissue obtained from 2 patients (2 females, aged 49 and 76 years) was used for collagen expression via RT-PCR. The adipose tissues from the 5 aforementioned patients were used for histological study.

The protocols used in this study were approved by the Research Ethics Committee (approval no. 2020-151) and the Human Studies Committee of Osaka Medical and Pharmaceutical University, Takatsuki City, Osaka, Japan. In accordance with the Declaration of Helsinki, informed consent was obtained from all patients prior to their involvement in this study.

### 2.2. General Histological and Immunohistological Studies

Paraffin blocks were prepared from formalin-fixed adipose tissues, and 3 µm serial cross-sections were prepared for histological staining. The first serial cross-sections from each of the paraffin blocks were stained with H&E to identify infiltrating lymphocytes. Azan Mallory staining was performed on the second cross-sections to identify fibrosis. H&E and Azan Mallory staining were performed in accordance with standard staining protocols. The fourth cross-sections were stained with toluidine blue to determine the MC distribution. Deparaffinized sections were immersed in 0.5% toluidine blue solution (pH 4.8) for approximately 15 min, fractionated with 0.5% glacial acetic acid solution, and then mounted after drying.

The third and fifth serial cross-sections were used to reveal the distribution of tryp-tase and chymase using anti-tryptase antibody (M7052, 1:800 dilution; Dako Denmark A/S, Glostrup, Denmark) and anti-chymase antibody (mouse monoclonal antibody against human MC chymase, 2D11G10D, 1:1000 dilution; a kind gift from Dr. Takeo Su-zuki, Katakura Industries Co., Ltd., Saitama, Japan), respectively. To evaluate the mesenchymal cellular components (such as fibroblasts and myofibroblasts) among adipose tis-sues, immunostaining for vimentin (1:100 dilution; Cell Signaling Technology, Danvers, MA, USA) and α-SMA (1:200 dilution; Dako) was performed on the sixth and seventh sections. The eighth section was used to reveal the distribution of PDFG-A using an anti–PDGF-A antibody (1:100 dilution, sc-9974, Santa Cruz Biotechnology Inc., Dallas, TX, USA). Immunohistological staining using the above-mentioned antibodies was performed in accordance with previously described protocols [18]. Deparaffinized sections were incubated with the respective antibodies overnight at 4 °C, followed by reaction with components from a labeled streptavidin–biotin peroxidase kit (LSAB^®^ System-HRP; Dako North America, Inc., Carpinteria, CA, USA) that included 3-amino-9-ethylcarbazole for color development. Finally, count staining for these sections was performed with hematoxylin, and the sections were then mounted with cover glasses. To determine the cell numbers in the two groups, the number of cells in each cross-section was counted at HPF (200) in the three densest areas (i.e., the hot spots), with the mean value then used for statistical analysis.

### 2.3. RT-PCR

Total RNA was extracted from frozen samples in accordance with the protocol pro-vided in a total RNA isolation kit (ISOGEN PB kit; Nippon Gene Co., Ltd., Tokyo, Japan). Total RNA (1 µg) was transcribed into cDNA using a SuperScript™ VILO™ cDNA Syn-thesis kit (Invitrogen™ Corp., Carlsbad, CA, USA), and mRNA levels were determined via RT-PCR using a Stratagene Mx3000P qPCR System (Agilent Technologies, Inc, Santa Clara, CA, USA) with Taq-Man™ (Applied Biosystems, Waltham, MA, USA) fluorogenic probes. RT-PCR primers and probes for collagen I, collagen III, TNF-α, PDGF-A, PDGFR-α, tryp-tase, chymase, and glyceraldehyde-3-phosphate dehydrogenase (GAPDH) were designed by Roche Diagnostics K.K. (Tokyo, Japan). The primers were as follows: 5′-caacagccgcttcacctac-3′ (forward) and 5′-caggctccggtgtgactc-3′ (reverse) for collagen I, 5′-tcctggtgctataggtccatct-3′ (forward) and 5′-caatcctcggtctccaggt-3′ (reverse) for collagen III, 5′-cagcctcttctccttcctgat-3′ (forward) and 5′-gccagagggctgattagaga-3′ (reverse) for TNF-α, 5′- cagtcagatccacagcatcc-3′ (forward) and 5′- caggctggtgtccaaagaat-3′ (reverse) for PDGF-A, 5′-tgcctgacattgaccctgt-3′ (forward) and 5′-ccgtctcaatggcactctct-3′ (reverse) for PDGFR-α, 5′-gatgctgagcctgctgct-3′ (forward) and 5′-gacgatacccgcttgctg-3′ (reverse) for tryptase, 5′-atccctcagacccaagagg-3′ (forward) and 5′-ggaagctggatctttattgagg-3′ (reverse) for chymase, and 5′-aatgtatcagttgtggatctgacc-3′ (forward) and 5′-gcttcactaccttcttgatgtcg-3′ (reverse) for GAPDH. The collagen I, collagen III, TNF-α, PDGF-A, PDGFR-α, chymase, and tryptase mRNA levels were normalized to that of GAPDH.

### 2.4. Statistical Analysis

All numerical data are expressed as the mean ± SEM. Significant differences between the mean values of the groups were evaluated via the Mann–Whitney U test. Pearson’s correlation coefficient was measured to test the linear relationship between two variables using linear regression analysis. A *p*-value of <0.05 was considered statistically significant.

## 3. Results

### 3.1. General Histological Examinations

**Figure 1:**
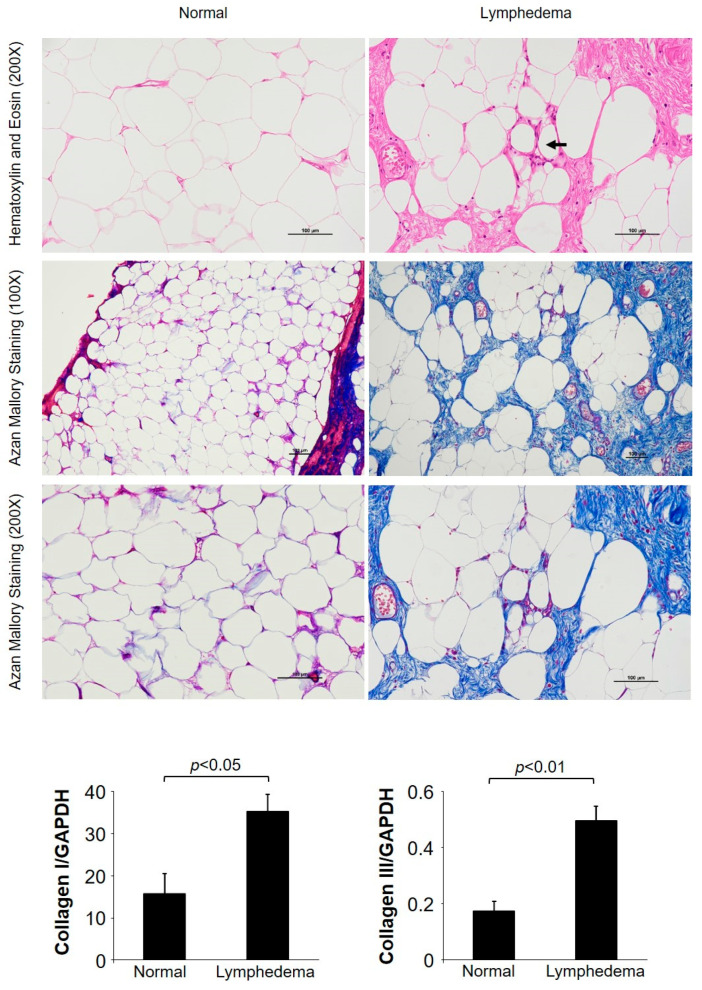
Representative images of Azan Mallory-stained and hematoxylin and eosin (H&E)-stained cross-sections of normal adipose tissue and tissue from patients with lymphedema are shown. The black arrows indicate the crown-like structure formed via the migration of inflammatory cells. The bar graphs show the gene expression levels of collagen I and collagen III in the normal adipose tissue and in tissue from patients with lymphedema.

Representative hematoxylin and eosin (H&E) staining and Azan Mallory staining images of lymphedema patient adipose tissue and normal control adipose tissue are shown in Figure 1. In control adipose tissue, there was no fibrosis, and the adipocyte structure was preserved. In contrast, in adipose tissue samples taken from lymphedema patients, we identified fibrosis-forming collagen bundles with trapped adipocytes of various sizes. The connective tissue was most prominent around blood vessels, and infiltration of inflammatory cells was also observed in these areas. Furthermore, crown-like structures with inflammatory cells (Figure 1, black arrow) were observed in the adipose tissue of lymphedema patients. Azan Mallory staining showed that adipocytes in the control group were surrounded by light-blue, thin, fibrotic interstitial tissue. In contrast, adipose tissue from lymphedema patients stained dark blue, and dark staining was especially prominent in the cytoskeleton and around blood vessels. Moreover, expression of the collagen I and collagen III genes was significantly elevated in adipose tissue from patients with lymphedema (*p* < 0.05) (Figure 1, bar graph).

**Figure 2:**
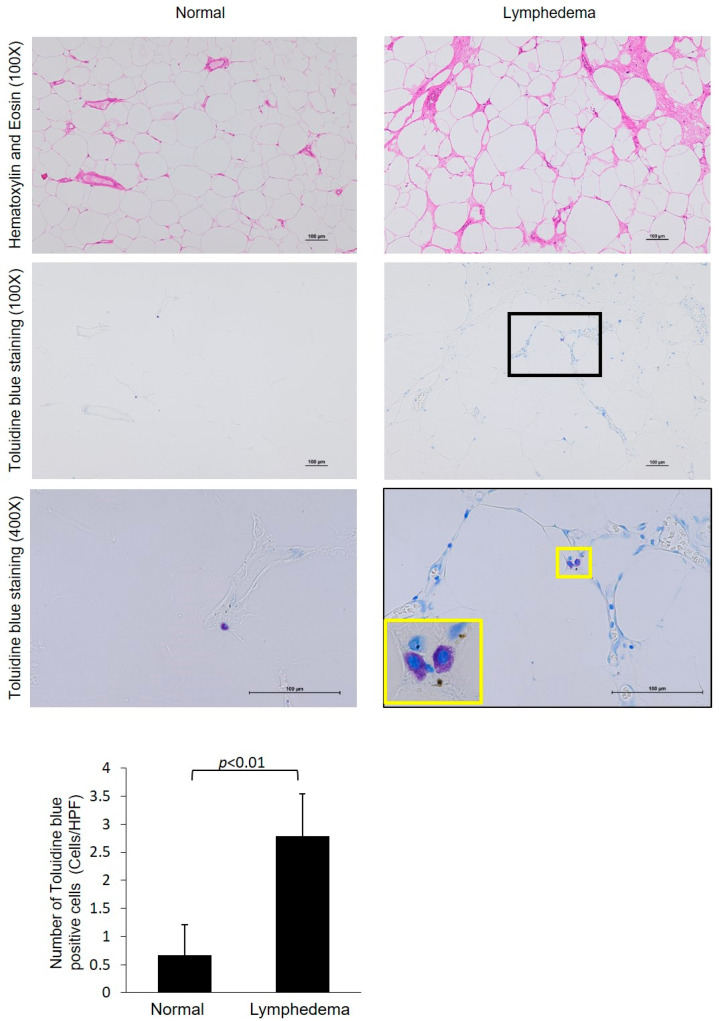
Representative images of toluidine blue staining and the calculated number of toluidine-positive cells in the normal adipose tissue and those from patients with lymphedema. The images below were higher-magnification images matched to the black frame. The cell indicated by the yellow frame was shown in the high-magnification image. The bar graph shows that the statistically significant increase in the amount of toluidine blue positivity was observed in lymphedema patients.

Representative toluidine blue staining images of lymphedema patient adipose tissue and normal control adipose tissue, as well as a comparison of the number of MCs between the two groups, are shown in Figure 2. A few toluidine blue-positive MCs were observed in the control adipose tissue, while marked accumulation of MCs was observed in the adipose tissue of lymphedema patients (Figure 2), and a statistically significant increase in the number of MCs was observed in lymphedema patients compared with control subjects (Figure 2, bar graph).

### 3.2. Identification of Mast Cell Type by Immunohistological Examinations

**Figure 3:**
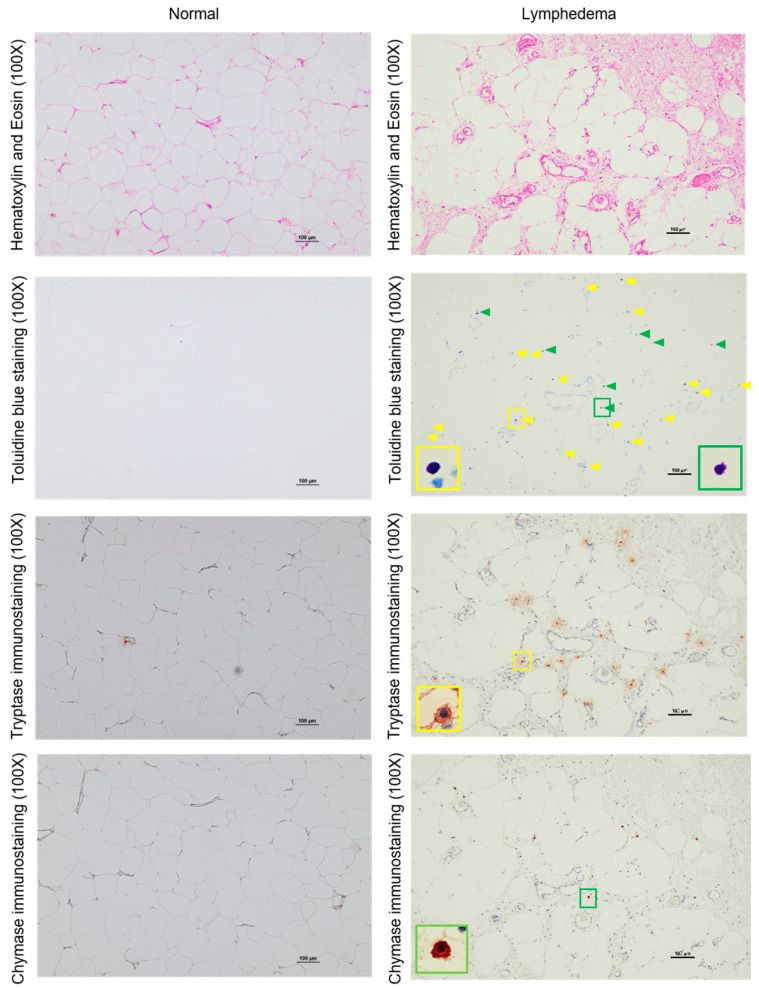
Representative images of H&E staining, toluidine blue staining, tryptase immunostaining and chymase immunostaining performed on serial sections of normal adipose tissue and the adipose tissue from patients with lymphedema are shown. For the sample with toluidine blue staining, yellow arrows indicate tryptase-positive MCs and green arrows indicate chymase-positive MCs. Cells indicated by yellow and green frames are shown in the high-magnification image.

**Figure 4:**
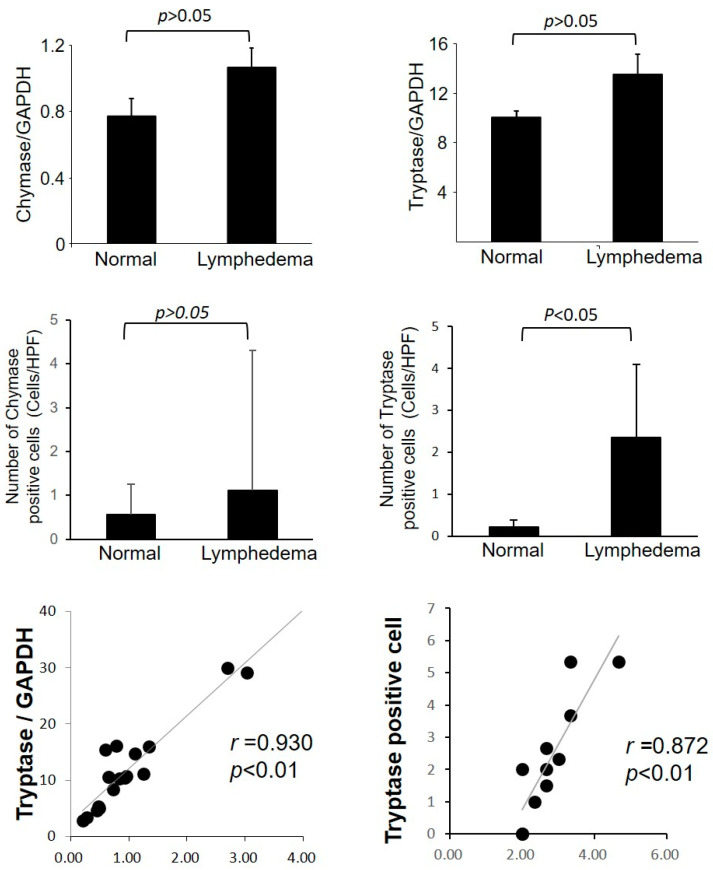
Representative bar graphs show the gene expression of chymase and tryptase as well as the calculated number of mast cells stained with chymase immunostaining (chymase-positive cells) and mast cells stained with tryptase immunostaining (tryptase-positive cells) in the normal adipose tissue and in the tissue of patients with lymphedema. Line graphs show the correlations between the gene expression of tryptase and TNF-α, as well as the calculated number of toluidine blue-positive cells and tryptase-positive cells in the adipose tissue from patients with lymphedema.

Figure 3 shows representative tryptase immunostaining images of lymphedema patient adipose tissue and normal control adipose tissue. The number of tryptase-positive cells and tryptase mRNA expression between the two groups is shown in Figure 4. The level of tryptase mRNA expression was generally higher in lymphedema patients. The expression of chymase mRNA also increased in lymphedema patients. As with the expression of MCs in control adipose tissue, the accumulation of tryptase-positive cells was low. However, numerous tryptase-positive cells accumulated in the adipose tissue of lymphedema patients. In comparison with control adipose tissue, the number of tryptase-positive cells was significantly higher in the adipose tissue of lymphedema patients (Figure 4). As indicated in the line graph, a strong positive correlation was found between the number of MCs and number of tryptase-positive cells. In contrast, although the number of chymase-positive cells was higher in lymphedema patients, the difference between lymphedema patient adipose tissue and normal control tissue was not significant (*p* > 0.05). With regard to the content of neutral proteases in secretory granules, two types of human MCs have been described. If MCs contain both tryptase and chymase in their secretory granules, these cells belong to the MCTC (tryptase-positive, chymase-positive) subtype, and if they contain only tryptase, they belong to the MCT (tryptase-positive, chymase-negative) subtype [19]. In this study, we observed a clear increase in the number of tryptase-positive cells compared to chymase-positive cells, indicating that the increase in the number of MCs in the adipose tissue of lymphedema patients may have been due to an increase in MCT-type MCs.

Tumor necrosis factor (TNF)-α is known for collagen accumulation and proliferation in fibroblasts. Linear regression analysis revealed a significant positive correlation between TNF-α gene expression and tryptase-positive cells in the adipose tissue of lymphedema patients (Figure 4).

### 3.3. Identification of Fibroblasts and Myofibroblasts in the Fibrotic Regions of Lymphedema

**Figure 5:**
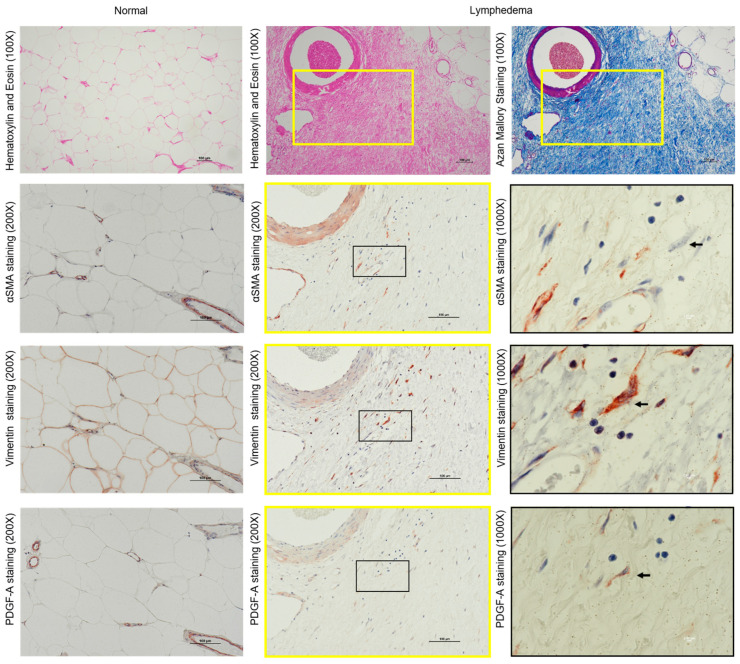
Representative α-SMA, vimentin, and PDGF-A immunostaining of serial cross-sections of normal adipose tissue and the adipose tissue from patients with lymphedema. These loci were matched to the yellow frames of H&E and Azan Mallory-stained sections shown in the upper column. The immunostained images surrounded by yellow frames are magnified 200×, and the black frames are magnified 1000×.

Representative images of vimentin and α–smooth muscle actin (α-SMA) immunostaining of serial sections of adipose tissue obtained from lymphedema patients are shown in Figure 5. As noted for the Azan Mallory staining shown in Figure 5, dark blue staining was observed in the adipose tissue of lymphedema patients surrounding the blood vessels, indicating extensive deposition of collagen and the presence of severe fibrosis. Vimentin-positive cells in fibrotic lesions of lymphedema patients were diffusely scattered in the adipose tissue, whereas a few α-SMA-positive cells were found in the serial sections alongside vimentin staining. These results indicate that fibroblasts are the non-adipocyte main cellular component in lymphedema tissues.

**Figure 6:**
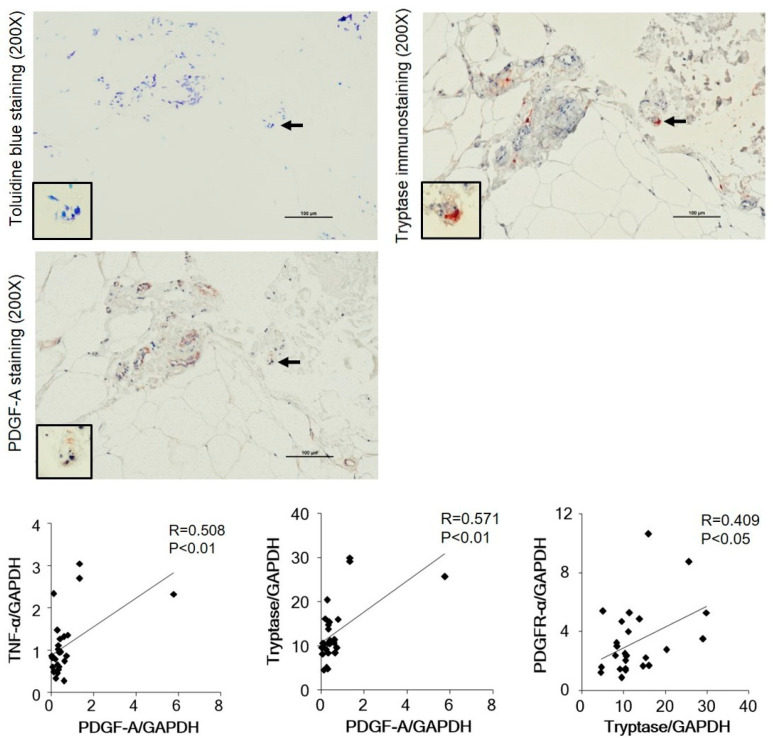
Representative toluidine blue, tryptase, and PDGF-A immunostaining of the serial sections of adipose tissue of lymphedema patients. The cell indicated by a black arrow is shown in high-power views. Mast cells identified via toluidine blue staining were located in the same position as the cells identified via tryptase staining, and some of the granules of the mast cells from the same site underwent PDGF-A immunostaining. Line graphs show the correlations between the gene expression of TNF-α and PDGF-A, tryptase and PDGF-A, as well as PDGFR-α and tryptase.

In our study, we also performed immunostaining of adipose tissue using antibodies against PDGF-A to investigate whether the phenomenon of PDGF-A-dependent fibroblast activation occurs in severely fibrotic regions of the adipose tissue of lymphedema patients. Immunostaining with PDGF-A revealed the presence of PDGFA-positive cells within these fibrotic areas. In the sections adjacent to vimentin staining, we found that cells positive for vimentin were also positive for PDGF-A (Figure 6, black arrows). Additionally, the results of vimentin, PDGF-A immunostaining, and toluidine blue staining on serial sections of adipose tissue from lymphedema patients revealed that MCT cells housed secretory granules that reacted positively to the anti-PDGF-A antibody (Figure 6).

## 4. Discussion

Lymphedema is a condition that develops after direct trauma, infection, surgery, or radiation therapy for cancers [1,2,3]. Once it has developed, it leads to progressive and irreversible changes. In secondary lymphedema, typically, the large collecting lymphatic vessels or nodes have been damaged due to lymph node resection and radiation therapy [20]. Insufficiency of lymphatic flow results in the accumulation of macromolecular proteins within the extracellular space throughout areas of local or downstream lymphatic obstruction [21]. The pathophysiology of lymph vessel obstruction involves a sustained inflammatory response that drives fluid deposition toward adipose expansion and fibrosis [22,23].

Increased expression of various cytokines and chemokines, as well as increased immune cell accumulation and activation, are typical features of the inflammatory response in lymphedema. Elevated CD4+ T cell counts in lymphedematous human tissue samples from affected limbs are correlated with lymphedema stage [24]. The number of CD4 cells that expressed Th2 cytokine IL4 and IL5 was increased in patients with lymphedema [8]. In addition to immune cell activation, lymphedema tissue has dramatically higher levels of pro-inflammatory cytokines TNF-α, IL-6, IL-8, and MCP-1, which can be used to measure the inflammatory response [25,26].

Analyses of clinical tissues have shown that lymphedema causes progressive collecting of lymphatic vessel fibrosis, smooth muscle cell proliferation, and lymphatic vessel luminal obstruction [5,6,27]. Fibro-adipose tissue deposition with increased deposition of type I and III collagen is also observed within the adipose tissue [27].

In healing wounds and fibrotic diseases of the lungs and skin in humans, increased numbers of MCs are observed in proliferating fibrous tissues [28,29,30]. Activation of MCs results in extracellular release of chemical mediators such as histamine, the serine proteinases tryptase, and chymase [31,32,33,34]. MCs are classified into two types according to the serine proteinases contained in the secretory granules. MCTC-type MCs contain both tryptase and chymase, and MCT-type MCs contain tryptase but not chymase [19]. MCT cells predominate in the alveoli, whereas MCTC cells predominate in the dermis and small intestinal submucosa. These mast cells are involved in the fibrosis of each organ. Haimanot et al. reported that increased chymase expression in the renal interstitium can induce renal fibrosis by activating transforming growth factor β (TGF-β) and angiotensin II signaling [35]. In lymphedema, towards the end of stages II and III, the extremities may not pit due to skin thickening and further deposition of adipose tissue. In the dermis of lymphedema patients, MCT cells have been identified as well as CD4-positive cells [23]. The number of CD4^+^ Th cells in the dermis and subcutaneous tissue is positively correlated with the stage of lymphedema [9]. MCT-type MCs are also present in lymphoid follicles. T-lymphocyte factors in the local environment are thought to facilitate the appearance of MCT cells [14]. This phenomenon is believed to be responsible for the appearance of MCT cells in inflamed areas [36]. In other words, environmental changes in local immunity due to lymphatic accumulation may contribute to the presence of MCT cells.

Another study performed by Sun et al. indicated that MCTC-type MCs are also involved in the thickened dermis, especially near the blood vessels and lymphatic vessels of the limb in patients with stage II or III lymphedema [15]. The accumulation of infiltrating microbes or specific antigen may induce IgE-mediated hypersensitivity. MCs facilitate the binding of IgE antibodies via FceRI receptors to the surfaces of MCs and proliferate a wide range of proteases, cytokines, and other mediators of inflammation. In particular, TNF-α and TGF-β are mast cell-associated mediators involved in fibroblast proliferation in IgE-dependent mast cell activation. In 1994, John et al. reported that mast cell supernatants’ ability to induce increased levels of collagen mRNA in mouse skin fibroblasts is markedly decreased by absorption with antibodies specific to either TNF-α and TGF-β and is eliminated entirely by absorption with antibodies against both cytokines in vitro [37]. Recently, Mehhara et al. reported that anti-Th2 therapy can reduce hyperkeratosis, epidermal proliferation, type II collagen deposition and the number of infiltrating mast cells in lymphedematous skin [8]. These results indicate that mast cells play a key role in the pathological process of skin fibrosis associated with lymphedema.

In this study, the number of MCT-type MCs was significantly increased in the fibrous region of adipose tissue of secondary lymphedema patients. Expression of the collagen I and III genes in the adipose tissue of secondary lymphedema was significantly increased compared with normal adipose tissues. In 1997, Jennifer et al. examined the effect of tryptase as a growth factor for human fibroblasts [38]. They cultured human lung fibroblasts with tryptase and found that tryptase stimulated an increase in collagen synthesis 48 h after addition to the cell culture. TGF-β induces profibrotic cells with contractile characteristics and is often considered the predominant profibrotic signal [39]. Jennifer et al. also incubated cells in the presence of both tryptase and a neutralizing antibody against TGF-β [38]. However, the antibody failed to change the response to tryptase, suggesting that other signals may also be involved in mast cell-induced fibrosis. Recently, PDGF was identified as another important profibrotic signal that binds the receptor tyrosine kinases PDGFRα and PDGFRβ [40]. In humans, PDGF-A is stimulated by TNF-α and promotes fibroblast proliferation. In this study, real-time polymerase chain reaction (RT-PCR) results showed a strong correlation between tryptase and TNF-α expression, as well as a strong correlation between TNF-α and PDGF-A and between tryptase and PDGF-A (Figure 6). These results agree with previous observations reported by Pulsson et al. and Battegay et al. that TNF-α increases the level of PDGF-A [41,42].

PDGF has been extensively studied as a key factor secreted by immune cells, including MCs [43]. In 2016, Nazari et al. studied the PDGF pathway as a possible mediator of MC modulation of mesenchymal stem cells (MSCs) [44]. They found that blockade of the PDGF pathway using a potent and selective PDGF-receptor kinase inhibitor (AG1296) attenuated MC granulate treatment-induced MSC migration and α-SMA and miR-145 downregulation. Their results suggest that PDGF secreted by MCs plays an important role in the de-differentiation of MSCs, promoting their proliferation and migration.

In this study, our results showed strong correlations between the expression of tryptase and PDGF-A and PDGFRα in adipose fibrosis of secondary lymphedema patients. Furthermore, vimentin and PDGF-A immunostaining performed on serial sections of adipose tissue obtained from lymphedema patients showed that MCT-type MCs contained secretory granules stained with anti-PDGF-A antibody (Figure 6). These results suggest that MC-derived tryptase plays a role in the fibrosis of adipose tissue due to lymphedema, either directly or in cooperation with other mediators.

### Limitation

This work focused on the histological distribution and degree of tryptase expression in adipose tissues of patients with lymphedema and controls without lymphedema. The data indicate that MCs express several mediators, which may change in patients with lymphedema. In this study, the sample size of the control group is small, which may affect the generalizability and validity of the results. Furthermore, we cannot reach a final conclusion until beneficial effects are obtained with tryptase-specific inhibitor treatment in animal models that resemble the pathophysiology of lymphedema.

## 5. Conclusions

In this study, the number of tryptase-positive MCs was markedly increased in the adipose tissue of secondary lymphedema patients. RT-PCR results showed a strong correlation between tryptase and TNF-α expression, as well as a strong correlation between TNF-α and PDGF-A, and between tryptase and PDGF-A. Furthermore, vimentin and PDGF-A immunostaining of serial sections of adipose tissue obtained from lymphedema patients showed that MCT-type MCs contained secretory granules stained with the anti–PDGF-A antibody. These results suggest that tryptase-positive MCs contribute to adipose fibrosis in secondary lymphedema through PDGF.

## Data Availability

The data that support the findings of this study are available on request from the corresponding author, Takashi Nuri (E-mail: takashi.nuri@ompu.ac.jp).
